# Molecular mechanisms of signaling via the docosanoid neuroprotectin D1 for cellular homeostasis and neuroprotection

**DOI:** 10.1074/jbc.R117.783076

**Published:** 2017-06-14

**Authors:** Aram Asatryan, Nicolas G. Bazan

**Affiliations:** From the Neuroscience Center of Excellence, School of Medicine, Louisiana State University Health New Orleans, New Orleans, Louisiana 70112-2223

**Keywords:** cell death, lipid signaling, neurodegenerative disease, NF-kB transcription factor, retina

## Abstract

Docosahexaenoic acid, enriched in the brain and retina, generates docosanoids in response to disruptions of cellular homeostasis. Docosanoids include neuroprotectin D1 (NPD1), which is decreased in the CA1 hippocampal area of patients with early-stage Alzheimer's disease (AD). We summarize here how NPD1 elicits neuroprotection by up-regulating c-REL, a nuclear factor (NF)-κB subtype that, in turn, enhances expression of BIRC3 (baculoviral inhibitor of apoptosis repeat-containing protein 3) in the retina and in experimental stroke, leading to neuroprotection. Elucidating the mechanisms of action of docosanoids will contribute to managing diseases, including stroke, AD, age-related macular degeneration, traumatic brain injury, Parkinson's disease, and other neurodegenerations.

## Introduction

A single cell zygote embarks on a venture that will, for the next 9 months, produce descendant cells with a colossal variety of shapes and functions. These differentiated cells, such as fibroblasts, astrocytes, neurons, the cells of the eyes, and others, then arrange into a distinct pattern to form the organs of our body. Some cells actively divide throughout their lives, although terminally differentiated cells like neurons and retinal pigment epithelial (RPE) ^2^ cells stay with us for a lifetime. These cells carry an enormous responsibility for the proper function of the organ of which they are a part, which requires well-sustained homeostasis. In 1926, Hans Selye first used the word “stress” in a biological context, referring to the non-specific response of the body to any demands placed upon it (1). He concluded that stress is a condition in which demand exceeds the regulatory capacity of an organism, and this excessive demand causes a disruption in homeostatic equilibrium. The central nervous system (CNS) plays a pivotal role in the stress-response mechanisms, and the consequences of disrupting homeostasis could potentially threaten neuronal or astrocytic cell survival.

## Formidable cellular alliance: RPE and photoreceptor cells

Neuroectodermally derived RPE cells form a single layer adjacent to the tip of photoreceptor outer segments and contribute to sustaining photoreceptor cell (PRC) homeostasis. RPE cells are the most active phagocytes in the body and, during daily shedding and phagocytosis of PRC outer segments, recycle visual cycle components and docosahexaenoic acid (DHA) in a circadian manner (2–5). Both PRCs and RPE cells constantly must manage the reactive oxygen species (ROS) that result from the oxygen-rich environment, high metabolic activity, high flux of polyunsaturated fatty acids (PUFAs) (*e.g.* omega-3 and omega-6), and energy from light. Therefore, RPE cells and PRCs are at a high risk for the uncompensated oxidative stress (UOS) that, in turn, could disrupt homeostasis (6, 7). Omega-3 and omega-6 PUFAs are necessary for cell function, are vital constituents of cell membranes, and play an important role in cell integrity and development. DHA, an essential omega-3 fatty acid family member, is retained and concentrated in PRCs and synaptic membranes, where it is esterified to phospholipids (8). DHA can be acquired through diet, as well as through elongation and desaturation of α-linoleic acid in hepatocytes. DHA is then esterified to the *sn*-2 position of phospholipids, mainly phosphatidylcholine (PC), and is packaged into lipoproteins and transported via the blood to the brain, retina, and other tissues. Thus, the liver supplies DHA to different tissues in the body (9–13). In the eye, esterified DHA is cleaved from lipoproteins by lipoprotein lipases at the choriocapillaries and is taken up at the basal side of RPE cells. DHA is then delivered to the PRC bodies (“inner segments”), whereupon it becomes incorporated into the phospholipids that subsequently are assembled into cellular membranes, most notably the “disc” membranes that constitute the PRC outer segments (14). The uptake of DHA is mediated by adiponectin receptor 1 (AdipoR1), a heptahelical transmembrane protein with a topology that is inverse to that of the G-proteins (13). Genetic deletion of this receptor leads to a drastic reduction of the following: (*a*) the ELOVL4-catalyzed synthesis of omega-3 very-long-chain polyunsaturated fatty acids (VLC-PUFAs); (*b*) the molecular species of phosphatidylcholine-containing VLC-PUFAs in the *sn*-1 of the glycerol backbone; and (*c*) the DHA residing in the *sn*-2 (13). All of this results in retinal degeneration, which presents itself in the form of a flecked retina resembling the human fundus albipunctatus with an unchanged vasculature. The flecked appearance is due mainly to an accumulation of anti-F4/80-positive cells (activated macrophages) beneath the RPE. Moreover, it has been reported recently that an *adipoR1* mutation (c.929A→G), which leads to one amino acid substitution (p.Y310C), co-segregates with a large family in autosomal dominant retinitis pigmentosa (adRP) (15). The p.Y310C mutation alters AdipoR1 folding and subcellular localization *in vitro*. In addition, its knockdown in a morpholino zebrafish model decreases the number of rod photoreceptors selectively, but not the cones; this is a distinctive phenotype of retinitis pigmentosa. Thus, *adipoR1* is a novel adRP-causing gene that plays a key function in rod development and conservation. There are several possible mechanisms that may explain why retinal degeneration occurs when PRCs fail to capture DHA: (*a*) rhodopsin function may be perturbed because phosphatidylcholine-containing VLC-PUFAs interact intimately with this protein; (*b*) a shortage of docosanoids may lead to a rapid activation and accumulation of macrophages beneath the RPE, perturbing the functional integrity of this cell and, in turn, affecting PRC viability; and (*c*) a shortage of mediators necessary for PRC and RPE cell integrity.

## DHA arrival to the brain and docosanoids

In the brain, DHA-containing PCs are cleaved by the lipoprotein lipase that resides on the luminal side of the endothelial cells of the neurovascular unit. Cleaved lipids are then transported to the astrocytes and then finally to the neurons. The exact mechanisms are currently being studied. Ultimately, DHA ends up enriched in phospholipids of both the synapses and dendrites.

Pre-clinical events in AD include neuroinflammation, dendritic spine damage, and synaptic dysfunction exacerbated by UOS, which leads to dementia. These events coincide with the decreased DHA content in the brain. A study of brains from early-stage AD patients (with neuronal loss) shows that the amount of DHA and its enzymatic derivative, neuroprotectin D1 (NPD1; 10*R*,17*S*-dihydroxy-docosa-4Z,7Z,11E,13E,15Z,19Z-hexaenoic acid), is decreased in the CA1 region of the hippocampus (16). NPD1 and other DHA-derived lipid mediators, such as resolvins D1–D5 and maresins, are known as docosanoids and foster overall inflammation resolution (17, 18).

Early responses to inflammation at the cellular or systemic level include the cleavage of DHA by phospholipase A_2_, and then its lipoxygenation by 15-lipoxygenase-1 into NPD1 (19, 20).

The neuroprotective bioactivity of NPD1 includes inflammatory modulating properties and anti-apoptotic features, both of which contribute to restoring disrupted homeostasis (20, 21). In RPE cells subjected to UOS, PRC phagocytosis elicits NPD1-specific protection that is not mediated by inert polystyrene particles (21).

Furthermore, NPD1 shifts β-amyloid precursor protein (βAPP) processing toward the non-amyloidogenic route and decreases amyloid-β42 (Aβ42) release. DHA, in turn, elicits an Aβ42-lowering effect both *in vitro* and *in vivo* (22, 23).

## Significance of bioactive lipids in ischemic stroke

Activation of *N*-methyl d-aspartate (NMDA) glutamate receptors occurs after stroke and in epilepsy and other neurological diseases (24). This activation is accompanied by an increase in membrane phospholipid hydrolysis with concomitant accumulation of free fatty acids (mainly DHA and arachidonic acid) and stearoyl-arachidonoyl-*sn*-glycerol (25) due to the rapid activation of phospholipases A_2_ and C. DHA administration (i.v.) improves behavioral deficit and reduces infarct volume and edema after experimental focal cerebral ischemia (26, 27). Enzymatic lipoxygenation of DHA in experimental ischemic stroke results in NPD1 formation, which then counteracts pro-inflammatory bioactivity and contributes to sustained neuroprotection (16, 28). The onset of ischemia causes damage to neurons, and one could argue that in rodent experimental models even after 8 h of reperfusion, accumulation of endogenous neuroprotective mediators (including NPD1) is not sufficient to cope with the resulting damage. Between 24 and 72 h after reperfusion, polymorphonuclear leukocytes migrate to the brain parenchyma, initially removing debris. However, because of their large numbers, these leukocytes induce inflammation and further promote injury, thus increasing stroke size. Continuous injection of NPD1 into the fourth ventricle (400 ng/48 h) after ischemic experimental stroke reduced stroke size in rats by 50% (28, 29), attenuated polymorphonuclear leukocyte infiltration, and inhibited ischemia induction of cyclooxygenase-2 (COX-2) and nuclear factor κB (NF-κB) (18, 28). The cerebral ischemic core is surrounded by an area called the penumbra, and due to decreased blood circulation, cell viability is sustained only for 2–4 h post-infarction; therefore, it is critical to protect the penumbra. There is a high correlation between reducing the size of the penumbra and neurological recovery (30).

The two molecular cell death conduits that have been shown to occur during cerebral ischemia are the intrinsic apoptotic pathway (release of cytochrome *c* from the mitochondrial inner membrane and activation of associated caspases-3 and -7) and the extrinsic apoptotic pathway, which are activated as a result of the death-domain-containing receptor occupancy, with subsequent caspase-8 activation (31–33). NPD1 counteracts this pro-apoptotic activity by promoting differential expression of Bcl-2 family proteins, up-regulating anti-apoptotic Bcl-2, Bcl-xL, and Bfl-1/A1, and by attenuating the expression of pro-apoptotic Bax, Bad, and Bid. Furthermore, NPD1 reduces the activation of caspase-3 induced by oxidative and proteotoxic stress (20, 34). It also rescues RPE cells from apoptosis triggered by the UOS that is induced by H_2_O_2_ plus tumor necrosis factor α (TNFα) (20). Apoptosis also takes place in non-neuronal cells, such as astrocytes, where caspase-independent mechanisms play an important role (35).

## Cell death: The point of no return

**Figure 1.**
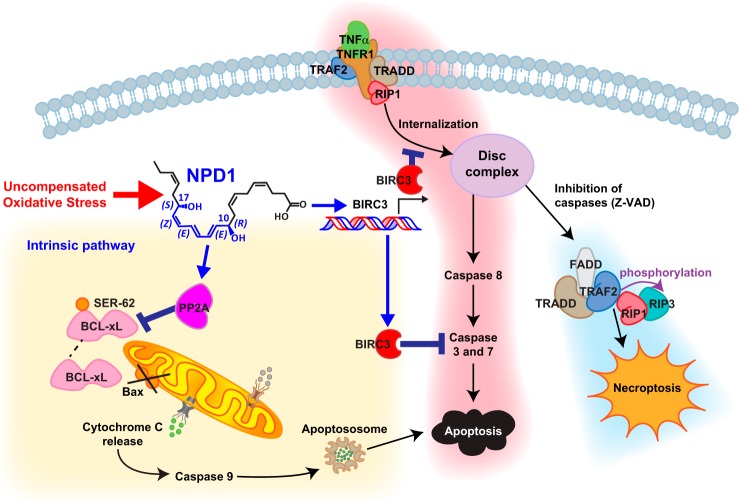
**NPD1 inhibits apoptosis during UOS.** Ligation of TNFR1 (highlighted in *pink*) stimulates the formation of complex I, which consists of TNFR1, TRADD, RIPK1, TNFR-associated factor 2 (*TRAF2*), BIRC2, and BIRC3. In the absence of BIRCs, RIP1 is deubiquitinated, which results in internalization of complex I, and procaspase-8 and RIP3 are integrated to form a new signaling complex (complex II or DISC). BIRCs suppress the formation of this complex, which acts as the activation platform for caspase-8, which induces cell death by the extrinsic pathway. Under conditions in which caspase activation is inhibited, RIP1 and RIP3 may be phosphorylated, thus forming a complex named necroptosome, which leads to cell death through a caspase-independent process known as necroptosis (highlighted in *blue*). UOS-triggered NPD1 exerts anti-apoptotic activity by increasing BIRC3 expression, resulting in subsequent augmentation of its anti-apoptotic effects. NPD1 also promotes dephosphorylation of Bcl-xL and promotes its heterodimerization with pro-apoptotic Bax, resulting in the consequent inactivation of this protein (highlighted in *yellow*). *Z-VAD*, benzyloxycarbonyl-VAD.

**Figure 2.**
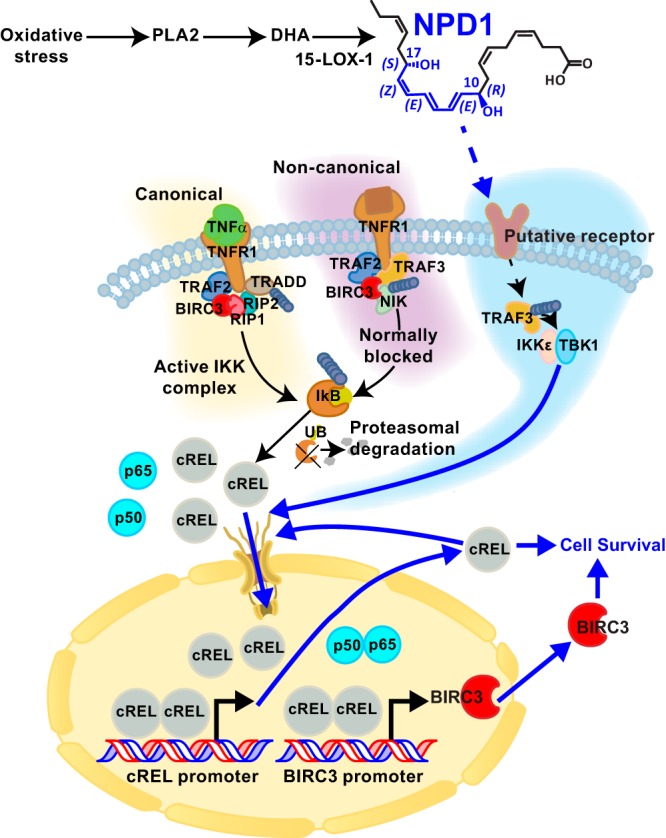
**Up-regulation of BIRC3 transcription by NPD1-dependent c-REL expression.** Stimulus-driven proteosomal degradation of IκB proteins is mediated by the IKK complex, which liberates NF-κB dimers and results in their further translocation into the nucleus and transcriptional activity. NF-κB signaling often is divided into two types of pathways. The canonical pathway (*yellow*) is induced by most physiological NF-κB stimuli and is represented here by TNFR1 signaling in an IKKβ- and NEMO-dependent manner, with resulting activation of RelA containing heterodimers. In contrast, the non-canonical pathway (*purple*) is stimulated by certain TNF family cytokines, such as CD40L and lymphotoxin-β, and it involves IKKα-mediated phosphorylation and generation of p52-RELB complexes in a NIK-dependent manner, which is subject to a complex regulation by TRAF3, BIRC3, and other ubiquitin (*UB*) ligases. Activation of both pathways could generate c-REL containing hetero- or homodimers. NPD1, which is synthesized in response to oxidative stress in the eye and brain, triggers the production of c-REL-containing dimers independent of both the canonical and non-canonical NF-κB pathways, possibly by modifying the c-REL TBK1/IKKϵ axis via activation of a putative receptor (*blue*).

The Nomenclature Committee on Cell Death proposes unified criteria for the definition of cell death and its different morphologies. Cell death is considered to be reversible until a first “point-of-no-return” checkpoint is passed (36). Cells can die in multiple ways, but by far the most studied forms of cell death are apoptosis and necrosis (37, 38). Newer cell death activation pathways have been described recently, including necroptosis (programmed necrosis), pyroptosis (caspase-1 activation), pyronecrosis (similar to pyroptosis but without the involvement of caspase-1), entosis (when one cell engulfs its living neighbor, also known as “cell cannibalism”), and others. Both necrosis and apoptosis are the main cell death pathways in brain ischemia-reperfusion injury. Cell apoptosis plays a critical role in regulation of cell homeostasis. In degenerative diseases, such as AD, Parkinson's disease, age-related macular degeneration, and stroke, dysregulation of apoptosis is a central event. Ligation of death receptors results in the formation of death-inducing signaling complex (DISC), activation of caspase-8, and subsequent cleavage and activation of caspases-3 and -7, with subsequent cell death (Fig. 1) (32). In an experimental model of RPE cell apoptosis, the TNFR1 death receptor ligand TNFα and H_2_O_2_ induce cell death (20, 39). Hydrogen peroxide completes a family of reactive oxygen species (ROS) that are formed from the partial reduction of oxygen (40). These compounds are formed continuously as by-products of aerobic metabolism, such as from reactions to drugs and environmental toxins, or when the levels of antioxidants are diminished, thus creating a condition of oxidative stress that ultimately contributes to cell death. ROS have been implicated in cancer, reperfusion injury, inflammatory diseases (such as multiple sclerosis), and aging. Although TNFα has been shown to be released by astrocytes and some neurons under basal conditions, its release is augmented during experimental ischemic stroke. After ligation to one of its receptors, TNFα forms a heterotrimer and, depending on the microenvironment, can cause apoptosis, necrosis, necroptosis, inflammation, or proliferation (Figs. 1 and 2). Binding of TNFα to TNFR1 (p55, CD120a) results in the formation of the so-called complex I (DISC), which is formed by the recruitment of receptor-interacting protein 1 (RIP1), TNF receptor-associated factor 2 (TRAF2), and the E3 ubiquitin ligases BIRC3 and BIRC2 by the TNFR type 1-associated DEATH domain (TRADD)-containing adaptor (41–43). This complex keeps pro-apoptotic pathways in check mainly by transcriptional activation of NF-κB and AP-1 (44–46). BIRC2, -3, and -4 harbor carboxyl-terminal RING finger domain with E3 ubiquitin ligase activity, which allows them to control cell fate. Both BIRC2 and BIRC3 ubiquitinate RIP1, and they are critical kinases in NF-κB activation (Fig. 1). In the absence of BIRC2 and BIRC3, RIP1 mediates both cell death and the activation of the caspase protein cascade (47, 48). NPD1 up-regulates BIRC3 expression 2–6 h after the initiation of oxidative stress (by introducing H_2_O_2_ and TNFα). In 15-lipoxygenase-1-deficient cells (which lack the ability to synthesize the lipid mediator NPD1) (19), only the addition of NPD1 increases the expression of *birc3* mRNA (39). Even though BIRC3 has not been shown to inhibit caspases-3 and -7 directly *in vivo*, growing evidence supports the idea that BIRC3 inhibits the activation of caspases. This can happen by polyubiquitination and subsequent proteosomal degradation of caspases, or it can occur indirectly by inhibiting other caspase activator mechanisms (45). Interestingly, the addition of NPD1 decreases oxidative and proteotoxic stress-triggered caspase-3 activation (20). Furthermore, NPD1 also suppresses activation of caspases-3 and -7 in RPE cells, and this effect is abolished in BIRC3-silenced RPE cells, suggesting that the bioactive lipid inhibition of caspases is mediated by BIRC3 (39). NPD1 affects BIRC2 expression slightly, thus canceling out the possibility of BIRC2-mediated caspase inhibition by NPD1. BIRC2 also is known as a weak inhibitor of caspases (49), and NPD1 also has been shown to be stereospecific since other lipid mediators, such as maresin, lipoxin-A4, and resolvin E1 (RvE1), and the two NPD1 stereoisomers RR-NPD1 and SS-NPD1 have not been shown to up-regulate BIRC3 significantly.

Once the ligated TNFR1 receptor is internalized, a second complex is formed upon deubiquitinated RIP1 recruitment to TRADD, Fas-associated protein with death domain (FADD), and pro-caspase-8 (forming complex II or DISC), which further activates caspase-8 and starts the cell death cascade (32). RIP1 has a fundamental role in cell death and survival homeostasis. Mice lacking RIP1 die within 3 days after birth. Additionally, cells defective in RIP1 are more sensitive to TNFα-induced death (50–52). NPD1 anti-apoptotic and caspase inhibitory effects involve RIP1, which is a ubiquitination target for BIRC3 (Fig. 1). NPD1 fails to prevent cell death in BIRC3-silenced RPE cells (39).

An inflammatory form of cell death, termed necroptosis, is involved in pathogen-induced and sterile inflammation and some neurodegenerative diseases (53). A multiprotein complex was recognized in TNFα-induced necroptosis after RIP3 was discovered in a complex with RIP1. As in the case of complex II, FADD and caspase-8 also were found to be a part of this new complex; it was called complex IIb or “necrosome” (48–49). Caspase-8 can inactivate RIP1 and RIP3 directly by cleaving them. The RIP1–RIP3 complex is the key to necroptosis (48, 49, 54). Caspase-8^−/−^ mice are not viable because of the active RIP1–RIP3 complex. Indeed, inhibition of RIP1 by necrostatin-1 abolishes both apoptosis and necrosis in RPE cells. Interestingly, the addition of the pan-caspase inhibitor benzyloxycarbonyl-VAD does not reduce either apoptotic or necrotic cell numbers considerably, supporting the involvement of the RIP1–RIP3 complex in cell death (39).

## Bioactive lipids modulate the transcriptome in inflammation and disease

A typical gene is surrounded by a high abundance and a wide variety of transcriptional regulators. A multitude of cis-regulatory elements per gene is convoluted by distal enhancers, core promoters, and by the high order of the spatial organization of the genetic elements (55, 56). The mechanisms of action for dietary fatty acids include the activation or suppression of DNA transcription. The earliest evidence of gene regulation by bioactive lipids comes from studies of peroxisome proliferator-activated receptors (PPAR), sterol regulatory element-binding protein 1 (SREBP1), fatty acid activation of Toll-like receptor 4 (TLR4), and several G protein-coupled receptors (*e.g.* GPR40, GPR120, GPR41, and GPR43) (57–59). Both eicosapentaenoic acid and DHA are natural activators of PPARs (60–62), and they both affect NF-κB signaling via the PPARα-mediated pathway (63). Moreover, NPD1 reduces Aβ42 peptide shedding by down-regulating β-secretase-1 (BACE1) via the PPARγ-dependent pathway, thereby shifting the cleavage of the βAPP holoenzyme from amyloidogenic to non-amyloidogenic (64).

## NF-κB: The good, the bad, or the ugly?

**Figure 3.**
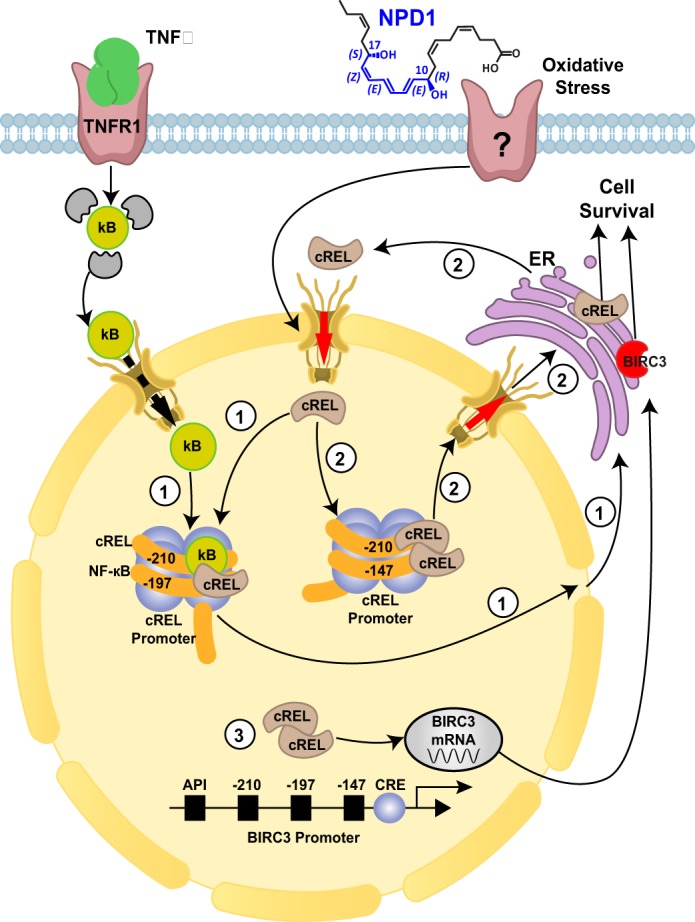
**Transcriptional regulation of c-REL by NPD1.** The activation of either the canonical/non-canonical NF-κB pathways or c-REL carboxyl-terminal phosphorylation by TBK1/IKKϵ results in accumulation of REL proteins in the nucleus. Depending on the conditions, homo- or heterodimers containing c-REL can form. (*1*) c-REL promoter ligation eventuates in c-REL protein synthesis, which reinforces the autoregulation loop, (*2*) by increasing the content of c-REL in the NF-κB dimers, and (*3*) c-REL-containing dimers bind to the BIRC3 promoter to increase its expression activity, which ensures cell survival. NPD1 augments this auto-regulation loop by shifting the balance toward c-REL homodimers to intensify the expression of both BIRC3 and c-REL genes with subsequent protein synthesis. This shift allows for the restoration of cell homeostasis and thus promotes survival. The endoplasmic reticulum (*ER*) is depicted for ease of understanding the protein synthesis step in the pathway.

The ability of BIRCs to regulate NF-κB signaling is central to cell survival, inflammation, and tumorigenesis (65–67). The role of BIRC2 and BIRC3 in response to TNFR1 activation has been studied in detail (68–70). Binding of trimeric TNFα recruits TRADD and then TRAF2, which in turn binds to BIRC2 and BIRC3 (Fig. 2). These two proteins are essential, positive regulators of the canonical pathway and are required for suppressing the constitutive activation of the non-canonical pathway. In turn, NF-κB induces the expression of BIRC2–4, thereby promoting NF-κB activation in a positive feedback loop (68). In the canonical pathway, the ligation of the TNF receptor by TNFα triggers a chain of events that leads to the following: the formation of a TAB1-TAK1-IKK complex; the phosphorylation, ubiquitination, and degradation of NF-κB inhibitors (IκBα, -β, -γ, and -ϵ); and the translocation of NF-κB dimers into the nucleus, followed by the transcriptional activation of a myriad of pro-inflammatory genes. In the non-canonical pathway, the ligation of CD40 and TNFRSF12A results in depletion of TRAF3, which is responsible for the NF-κB-inducible kinase (NIK) proteosomal degradation. NIK phosphorylates IκB kinase α (IKKα) at Ser-176 and Ser-180 and NF-κB2 at Ser-866 and Ser-870. This results in recruitment of IKKα heterodimers, further phosphorylation, and cleavage of NF-κB2 into the mature p52 form, which then dimerizes with another NF-κB member, RelB. This complex translocates into the nucleus and activates the transcription of genes (Fig. 2) (65). BIRC3 activates both the canonical and non-canonical NF-κB pathways (41, 43, 68, 69, 71). Although NF-κB family members RelA (p65), RelB, and c-REL are synthesized in their mature forms, carboxyl-terminal ankyrin repeats need to be cleaved off p100 and p105 to mature into p50 and p52. Rels contain an amino-terminal REL homology domain, which is necessary for dimerization and DNA binding. All these subtypes make homodimers and heterodimers in order to diversify the ways they modulate multiple signaling pathways.

c-*rel* is the cellular analog of the v-rel oncogene, a product of the avian reticuloendotheliosis virus strain T. The *rel* gene encodes a 587-amino acid protein, which binds to the GGGCTTTCC consensus sequence (72). NPD1 increases *birc3* promoter activity 4 and 6 h after stimulation with H_2_O_2_ and TNFα. Three cis-elements, located between −210 and −147 upstream of the transcriptional start site of the *birc3* promoter, turned out to be critical in NPD1-mediated c-*rel* transcriptional regulation. The *birc3* promoter has three NF-κB-binding sites (κB1, κB2, and κB3), and the deletion of two or more sites ablates NF-κB activation (73–75). The c-*rel* promoter has also been shown to have similar NF-κB-binding sites (76, 77). Indeed, our studies have shown that point mutations in two out of three binding sequences abolish the c-*rel* transcriptional regulation mediated by NPD1 (39). Oscillations in NF-κB signaling have been known for some time (78); however, it was unclear whether c-REL regulates its target genes in a similar manner. We showed previously that UOS triggers NPD1 synthesis, which induces c-REL mobilization into the nucleus as early as 2 h after the application of oxidative stress (39). c-REL mobilization follows a certain oscillatory pattern that returns to its peak 6 h after oxidative stress damage occurs. Initial mobilization increases c-REL expression by an auto-regulatory loop, which further augments transcriptional activity (Fig. 3). Interestingly, NPD1 enhances co-immunoprecipitation of c-REL-containing homodimers in RPE cells following an oxidative stress stimulus (39).

It has been shown that transcription of the *relB* gene is regulated by NF-κB (77, 79). Silencing of c-REL abolishes both *birc3* and *relB* gene expression, suggesting that the non-canonical pathway of NF-κB might be involved in c-REL-governed transcriptional modulation by NPD1. One could argue that the effects of NPD1 could potentially be the result of repressed canonical NF-κB-pathway activation. However, NPD1 phosphorylation of NF-κB inhibitors was not affected 2 h after oxidative stress induction; moreover, the total protein levels of both IκBα and Iκβ were reduced most likely due to proteosomal degradation (39). The lack of changes in phosphorylation suggests that IκBα and IκBβ regulation is governed by other mechanisms, such as mono- or polyubiquitination and degradation of these proteins.

The c-REL carboxyl terminus is composed of two transactivation domains (TAD1 and TAD2) separated by a transactivation REL inhibitory domain (RID) (80). It is extremely negatively charged and contains multiple serine residues. This suggests that the DNA transactivation ability of c-REL is regulated by post-translational modification. Indeed, the TBK1-IKKϵ complex phosphorylates the carboxyl terminus of c-REL, resulting in dissociation from IκBα (Fig. 2) (81). It also has been shown that Ser-471 phosphorylation and interaction with the region spanning amino acids 456–540 seem to be crucial for c-REL binding to NIK. Mutations of this region disrupt binding (82). NPD1 can modify the phosphorylation state of PP2A (83), and it also has been shown to alter AKT kinase activity (84, 85). It is possible that these kinases are responsible for the post-translational modification and regulation of c-REL by NPD1. c-REL knock-out (KO) mice have been shown to have immune system deficiencies, particularly during lymphocyte proliferation and activation. c-REL also plays a role in memory and learning (86), but one of the most notable roles of c-REL is neuroprotection. c-REL-containing dimers reduce neuronal susceptibility to anoxia (87) and protect against human islet cell death *in vitro* (88). Induction of anoxia both *in vitro* (via oxygen glucose deprivation of cortical neurons) and *in vivo* (using a model of cerebral ischemia) results in a reduction of nuclear RelA/c-REL and RelA/p50 dimers, whereas levels of p50/c-REL dimers remain unchanged. This neuroprotective effect perhaps is due to the effects on the anti-apoptotic protein Bcl-xL (87, 89, 90). Interestingly, NPD1 modulates the expression of Bcl-2 and Bcl-xL (19, 83). By promoting the dephosphorylation of Bcl-xL and its heterodimerization with pro-apoptotic Bax, NPD1 contributes to inactivation of the Bax protein (Fig. 1) (83). The anti-apoptotic effects of c-REL also can be explained, at least in part, by its effects on the anti-apoptotic BIRC2 and BIRC3; c-REL is a positive regulator of these two proteins (88).

Reactive oxygen species, as well as the peroxidation products of lipids, proteins, and DNA, create an inflammatory milieu during ischemia-reperfusion that initiates a neuronal cell death cascade. DHA, however, improves recovery after ischemia-reperfusion (26, 28). DHA administration, following middle cerebral artery occlusion in a rat model, results in c-REL translocation, BIRC3 protein expression, and neurological recovery (39). This resulting c-REL translocation is augmented with a subsequent increase in BIRC3 expression.

## Concluding remarks

Essential fatty acids have a variety of signaling mechanisms and cell functions. DHA, for example, in addition to being an important acyl chain of membrane phospholipids, influences homeostatic cellular responses via its biologically active derivatives, the docosanoids (*e.g.* NPD1). DHA is endowed richly in the brain and retina. An example of its critical meaning for cell function evolves from the finding that the genetic ablation of adiponectin receptor 1 shuts off the uptake of DHA in PRCs and leads to the death of these cells and to retinal degeneration (13). Moreover, it was found that a single amino acid mutation in this receptor is causative of autosomal dominant retinitis pigmentosa (91).

In conclusion, we summarized in this minireview the effects of the essential fatty acid family member DHA and its bioactive derivative NPD1 in the context of a specific target of gene regulation. We also described the mechanism of a pathway of regulation by a bioactive lipid that has a significant impact on cellular homeostasis. The induction of downstream pathways by NPD1 results in activation of pro-survival genes, suppression of pro-apoptotic genes, fostering of a pro-inflammatory milieu at the cellular level, and terminal differentiation of RPE and neuronal cells. The organizational and functional complexity of the brain raises new questions regarding how the cellular events described here operate in response to disrupted homeostasis to attain neuroprotection in pathological conditions. Understanding the molecular mechanisms of action of dietary essential fatty acids will lead to effective treatments of diseases and conditions such as stroke, Alzheimer's disease, age-related macular degeneration, Parkinson's disease, and other retinal and neurodegenerative diseases.

## Footnotes

2 The abbreviations used are:

RPE: retinal pigment epithelial
DHA: docosahexaenoic acid
AD: Alzheimer's disease
PRC: photoreceptor cell
UOS: uncompensated oxidative stress
ROS: reactive oxygen species
DISC: death-inducing signaling complex
PC: phosphatidylcholine
adRP: in autosomal dominant retinitis pigmentosa
AdipoR1: adiponectin receptor 1
VLC: very long-chain
βAPP: β-amyloid precursor protein
Aβ42: amyloid-β42
PPAR: peroxisome proliferator-activated receptor
NIK: NF-κB-inducible kinase
IKK: IκB kinase
TNFR: TNF receptor
TRADD: TNFR-associated via death domain
BIRC: baculoviral inhibitor of apoptosis repeat-containing protein.

## References

[1] Selye H. (1998) A syndrome produced by diverse nocuous agents. 1936. J. Neuropsychiatry Clin. Neurosci. 10, 230–231 9722327 10.1176/jnp.10.2.230a

[2] Kolko M., Wang J., Zhan C., Poulsen K. A., Prause J. U., Nissen M. H., Heegaard S., and Bazan N. G. (2007) Identification of intracellular phospholipases A2 in the human eye: involvement in phagocytosis of photoreceptor outer segments. Invest. Ophthalmol. Vis. Sci. 48, 1401–1409 17325189 10.1167/iovs.06-0865

[3] LaVail M. M. (1980) Circadian nature of rod outer segment disc shedding in the rat. Invest. Ophthalmol. Vis. Sci. 19, 407–411 7358492

[4] Bazan N. G., Calandria J. M., and Serhan C. N. (2010) Rescue and repair during photoreceptor cell renewal mediated by docosahexaenoic acid-derived neuroprotectin D1. J. Lipid Res. 51, 2018–2031 20382842 10.1194/jlr.R001131 PMC2903812

[5] Bok D. (1993) The retinal pigment epithelium: a versatile partner in vision. J. Cell Sci. Suppl. 17, 189–195 8144697 10.1242/jcs.1993.supplement_17.27

[6] Bazan N. (2006) Cell survival matters: docosahexaenoic acid signaling, neuroprotection and photoreceptors. Trends Neurosci. 29, 263–271 16580739 10.1016/j.tins.2006.03.005

[7] Bazan N. (2007) Homeostatic regulation of photoreceptor cell integrity: significance of the potent mediator neuroprotectin D1 biosynthesized from docosahexaenoic acid: the Proctor Lecture. Invest. Ophthalmol. Vis. Sci. 48, 4866–4881 17962433 10.1167/iovs.07-0918

[8] Bazan N. G., Silvia di Fazio Escalante M., Careaga M. M., and Bazan H. E. P. (1982) High content of 22:6 docosahexaenoate and active [2-^3^H]glycerol metabolism of phosphatidic acid from photoreceptor membranes. Biochim. Biophys. Acta 712, 702–706 6215065 10.1016/0005-2760(82)90301-0

[9] Bazan N. G., Birkle D. L., and Reddy T. S. (1985) in Retinal Degeneration: Experimental and Clinical Studies (LaVail M. M., Anderson G., and Hollyfield J., eds) pp. 159–187, Alan R. Liss, Inc., New York

[10] Scott B. L., and Bazan N. G. (1989) Membrane docosahexaenoate is supplied to the developing brain and retina by the liver. Proc. Natl. Acad. Sci. U.S.A. 86, 2903–2907 2523075 10.1073/pnas.86.8.2903 PMC287028

[11] Rapoport S. I., Rao J. S., and Igarashi M. (2007) Brain metabolism of nutritionally essential polyunsaturated fatty acids depends on both the diet and the liver. Prostaglandins Leukot. Essent. Fatty Acids 77, 251–261 18060754 10.1016/j.plefa.2007.10.023 PMC2725409

[12] Astarita G., Jung K.-M., Berchtold N. C., Nguyen V. Q., Gillen D. L., Head E., Cotman C. W., and Piomelli D. (2010) Deficient liver biosynthesis of docosahexaenoic acid correlates with cognitive impairment in Alzheimer's disease. PLoS One 5, e12538 20838618 10.1371/journal.pone.0012538 PMC2935886

[13] Rice D. S., Calandria J. M., Gordon W. C., Jun B., Zhou Y., Gelfman C. M., Li S., Jin M., Knott E. J., Chang B., Abuin A., Issa T., Potter D., Platt K. A., and Bazan N. (2015) Adiponectin receptor 1 conserves docosahexaenoic acid and promotes photoreceptor cell survival. Nat. Commun. 6, 6228 25736573 10.1038/ncomms7228 PMC4351799

[14] Bazan N. G., Gordon W. C., and Rodriguez de Turco E. B. (1992) Docosahexaenoic acid uptake and metabolism in photoreceptors: retinal conservation by an efficient retinal pigment epithelial cell-mediated recycling process. Adv. Exp. Med. Biol. 318, 295–306 1386177 10.1007/978-1-4615-3426-6_26

[15] Xu M., Eblimit A., Wang J., Li J., Wang F., Zhao L., Wang X., Xiao N., Li Y., Wong L. J., Lewis R. A., and Chen R. (2016) ADIPOR1 is mutated in syndromic retinitis pigmentosa. Hum. Mutat. 37, 246–249 26662040 10.1002/humu.22940 PMC5383450

[16] Lukiw W. J., Cui J.-G., Marcheselli V. L., Bodker M., Botkjaer A., Gotlinger K., Serhan C. N., and Bazan N. G. (2005) A role for docosahexaenoic acid-derived neuroprotectin D1 in neural cell survival and Alzheimer disease. J. Clin. Invest. 115, 2774–2783 16151530 10.1172/JCI25420 PMC1199531

[17] Serhan C. N. (2017) Treating inflammation and infection in the 21st century: new hints from decoding resolution mediators and mechanisms. FASEB J. 31, 1273–1288 28087575 10.1096/fj.201601222R PMC5349794

[18] Serhan C. N., Dalli J., Colas R. A., Winkler J. W., and Chiang N. (2015) Protectins and maresins: new pro-resolving families of mediators in acute inflammation and resolution bioactive metabolome. Biochim. Biophys. Acta 1851, 397–413 25139562 10.1016/j.bbalip.2014.08.006 PMC4324013

[19] Calandria J. M., Marcheselli V. L., Mukherjee P. K., Uddin J., Winkler J. W., Petasis N. A., and Bazan N. G. (2009) Selective survival rescue in 15-lipoxygenase-1-deficient retinal pigment epithelial cells by the novel docosahexaenoic acid-derived mediator neuroprotectin D1. J. Biol. Chem. 284, 17877–17882 19403949 10.1074/jbc.M109.003988 PMC2719426

[20] Mukherjee P. K., Marcheselli V. L., Serhan C. N., and Bazan N. G. (2004) Neuroprotectin D1: a docosahexaenoic acid-derived docosatriene protects human retinal pigment epithelial cells from oxidative stress. Proc. Natl. Acad. Sci. U.S.A. 101, 8491–8496 15152078 10.1073/pnas.0402531101 PMC420421

[21] Mukherjee P. K, Marcheselli V. L, de Rivero Vaccari J. C, Gordon W. C., Jackson F. E., and Bazan N. G. (2007) Photoreceptor outer segment phagocytosis selectively attenuates oxidative stress-induced apoptosis with concomitant neuroprotectin D1 synthesis. Proc. Natl. Acad. Sci. U.S.A. 104, 13158–13163 17670935 10.1073/pnas.0705963104 PMC1941804

[22] Sahlin C., Pettersson F. E., Nilsson L. N., Lannfelt L., and Johansson A. S. (2007) Docosahexaenoic acid stimulates non-amyloidogenic APP processing resulting in reduced Aβ levels in cellular models of Alzheimer's disease. Eur. J. Neurosci. 26, 882–889 17714184 10.1111/j.1460-9568.2007.05719.x

[23] Oksman M., Iivonen H., Hogyes E., Amtul Z., Penke B., Leenders I., Broersen L., Lütjohann D., Hartmann T., and Tanila H. (2006) Impact of different saturated fatty acid, polyunsaturated fatty acid and cholesterol containing diets on β-amyloid accumulation in APP/PS1 transgenic mice. Neurobiol. Dis. 23, 563–572 16765602 10.1016/j.nbd.2006.04.013

[24] Stark D. T., and Bazan N. G. (2011) Synaptic and extrasynaptic NMDA receptors differentially modulate neuronal cyclooxygenase-2 function, lipid peroxidation, and neuroprotection. J. Neurosci. 31, 13710–13721 21957234 10.1523/JNEUROSCI.3544-11.2011 PMC3197234

[25] Bazán N. G. Jr. (1970) Effects of ischemia and electroconvulsive shock on free fatty acid pool in the brain. Biochim. Biophys. Acta 218, 1–10 5473492 10.1016/0005-2760(70)90086-x

[26] Belayev L., Khoutorova L., Atkins K. D., Eady T. N., Hong S., Lu Y., Obenaus A., and Bazan N. G. (2011) Docosahexaenoic acid therapy of experimental ischemic stroke. Transl. Stroke Res. 2, 33–41 21423332 10.1007/s12975-010-0046-0 PMC3037476

[27] Eady T. N., Belayev L., Khoutorova L., Atkins K. D., Zhang C., and Bazan N. G. (2012) Docosahexaenoic acid signaling modulates cell survival in experimental ischemic stroke penumbra and initiates long-term repair in young and aged rats. PLoS ONE 7, e46151 23118851 10.1371/journal.pone.0046151 PMC3484151

[28] Marcheselli V. L., Hong S., Lukiw W. J., Tian X. H., Gronert K., Musto A., Hardy M., Gimenez J. M., Chiang N., Serhan C. N., and Bazan N. G. (2003) Novel docosanoids inhibit brain ischemia-reperfusion-mediated leukocyte infiltration and pro-inflammatory gene expression. J. Biol. Chem. 278, 43807–43817 12923200 10.1074/jbc.M305841200

[29] Hong S. H., Belayev L., Khoutorova L., Obenaus A., and Bazan N. (2014) Docosahexaenoic acid confers enduring neuroprotection in experimental stroke. J. Neurol. Sci. 338, 135–141 24433927 10.1016/j.jns.2013.12.033 PMC3943637

[30] Guadagno J. V., Calautti C., and Baron J. C. (2003) Progress in imaging stroke: emerging clinical applications. Br. Med. Bull. 65, 145–157 12697622 10.1093/bmb/65.1.145

[31] Reubold T. F., and Eschenburg S. (2012) A molecular view on signal transduction by the apoptosome. Cell. Signal. 24, 1420–1425 22446004 10.1016/j.cellsig.2012.03.007

[32] Wang L., Yang J. K., Kabaleeswaran V., Rice A. J., Cruz A. C., Park A. Y., Yin Q., Damko E., Jang S. B., Raunser S., Robinson C. V., Siegel R. M., Walz T., and Wu H. (2010) The Fas-FADD death domain complex structure reveals the basis of DISC assembly and disease mutations. Nat. Struct. Mol. Biol. 17, 1324–1329 20935634 10.1038/nsmb.1920 PMC2988912

[33] Hengartner M. (2000) DNA destroyers. Nature 407, 770–776 11048727 10.1038/35037710

[34] Calandria J. M., Mukherjee P. K., de Rivero Vaccari J. C., Zhu M., Petasis N. A., and Bazan N. G. (2012) Ataxin-1 poly(Q)-induced proteotoxic stress and apoptosis are attenuated in neural cells by docosahexaenoic acid-derived neuroprotectin D1. J. Biol. Chem. 287, 23726–23739 22511762 10.1074/jbc.M111.287078 PMC3390647

[35] Broughton B. R., Reutens D. C., and Sobey C. G. (2009) Apoptotic mechanisms after cerebral ischemia. Stroke 40, e331–e339 19182083 10.1161/STROKEAHA.108.531632

[36] Kroemer G., Galluzzi L., Vandenabeele P., Abrams J., Alnemri E. S., Baehrecke E. H., Blagosklonny M. V., El-Deiry W. S., Golstein P., Green D. R., Hengartner M., Knight R. A., Kumar S., Lipton S. A., Malorni W., et al (2009) Classification of cell death: recommendations of the Nomenclature Committee on Cell Death. Cell Death Differ. 16, 3–11 18846107 10.1038/cdd.2008.150 PMC2744427

[37] Krysko D. V., Vanden Berghe T., D'Herde K., and Vandenabeele P. (2008) Apoptosis and necrosis: Detection discrimination and phagocytosis. Methods 44, 205–221 18314051 10.1016/j.ymeth.2007.12.001

[38] Mattson M. P., and Bazan N. (2012) Basic Neurochemistry: Molecular, Cellular and Medical Aspects, 8th Ed., Chapter 37, pp. 663–676, Elsevier Inc. Academic Press, Cambridge, MA

[39] Calandria J. M., Asatryan A., Balaszczuk V., Knott E. J., Jun B. K., Mukherjee P. K., Belayev L., and Bazan N. G. (2015) NPD1-mediated stereoselective regulation of BIRC3 expression through c-REL is decisive for neural cell survival. Cell Death Differ. 22, 1363–1377 25633199 10.1038/cdd.2014.233 PMC4495360

[40] Turrens J. (2011) Mitochondrial formation of reactive oxygen species. J. Physiol. 8, 695–708 10.1113/jphysiol.2003.049478 PMC2343396 14561818

[41] Mace P. D., Smits C., Vaux D. L., Silke J., and Day C. L. (2010) Asymmetric recruitment of cIAPs by TRAF2. J. Mol. Biol. 400, 8–15 20447407 10.1016/j.jmb.2010.04.055

[42] Cabal-Hierro L., and Lazo P. S. (2012) Signal transduction by tumor necrosis factor receptors. Cell. Signal. 24, 1297–1305 22374304 10.1016/j.cellsig.2012.02.006

[43] Vince J. E., Pantaki D., Feltham R., Mace P. D., Cordier S. M., Schmukle A. C., Davidson A. J., Callus B. A., Wong W. W., Gentle I. E., Carter H., Lee E. F., Walczak H., Day C. L., Vaux D. L., and Silke J. (2009) TRAF2 must bind to cellular inhibitors of apoptosis for tumor necrosis factor (TNF) to efficiently activate NF-κB and to prevent TNF-induced apoptosis. J. Biol. Chem. 284, 35906–35915 19815541 10.1074/jbc.M109.072256 PMC2791019

[44] Liu Z. G., Hsu H., Goeddel D. V., and Karin M. (1996) Dissection of TNF receptor 1 effector functions: JNK activation is not linked to apoptosis while NF-κB activation prevents cell death. Cell 87, 565–576 8898208 10.1016/s0092-8674(00)81375-6

[45] Gyrd-Hansen M., and Meier P. (2010) IAPs: from caspase inhibitors to modulators of NF-κB, inflammation and cancer. Nat. Rev. Cancer 10, 561–574 20651737 10.1038/nrc2889

[46] Sullivan P. G., Bruce-Keller A. J., Rabchevsky A. G., Christakos S., Clair D. K., Mattson M. P., and Scheff S. W. (1999) Exacerbation of damage and altered NF-κB activation in mice lacking tumor necrosis factor receptors after traumatic brain injury. J. Neurosci. 19, 6248–6256 10414954 10.1523/JNEUROSCI.19-15-06248.1999 PMC6782813

[47] Feoktistova M., Geserick P., Kellert B., Dimitrova D. P., Langlais C., Hupe M., Cain K., MacFarlane M., Häcker G., and Leverkus M. (2011) cIAPs block Ripoptosome formation, a RIP1/caspase-8 containing intracellular cell death complex differentially regulated by cFLIP isoforms. Mol Cell. 43, 449–463 21737330 10.1016/j.molcel.2011.06.011 PMC3163271

[48] Tenev T., Bianchi K., Darding M., Broemer M., Langlais C., Wallberg F., Zachariou A., Lopez J., MacFarlane M., Cain K., and Meier P. (2011) The Ripoptosome, a signaling platform that assembles in response to genotoxic stress and loss of IAPs. Mol. Cell 43, 432–448 21737329 10.1016/j.molcel.2011.06.006

[49] Burke S. P., Smith L., and Smith J. B. (2010) cIAP1 cooperatively inhibits procaspase-3 activation by the caspase-9 apoptosome. J. Biol. Chem. 285, 30061–30068 20667824 10.1074/jbc.M110.125955 PMC2943263

[50] Bertrand M. J., Milutinovic S., Dickson K. M., Ho W. C., Boudreault A., Durkin J., Gillard J. W., Jaquith J. B., Morris S. J., and Barker P. A. (2008) cIAP1 and cIAP2 facilitate cancer cell survival by functioning as E3 ligases that promote RIP1 ubiquitination. Mol. Cell 30, 689–700 18570872 10.1016/j.molcel.2008.05.014

[51] Lee T. H., Huang Q., Oikemus S., Shank J., Ventura J. J., Cusson N., Vaillancourt R. R., Su B., Davis R. J., and Kelliher M. A. (2003) The death domain kinase RIP1 is essential for tumor necrosis factor α signaling to p38 mitogen-activated protein kinase. Mol. Cell. Biol. 23, 8377–8385 14585994 10.1128/MCB.23.22.8377-8385.2003 PMC262426

[52] Christofferson D. E., Li Y., Hitomi J., Zhou W., Upperman C., Zhu H., Gerber S. A., Gygi S., and Yuan J. (2012) A novel role for RIP1 kinase in mediating TNFα production. Cell Death Dis. 3, e320 22695613 10.1038/cddis.2012.64 PMC3388236

[53] Moriwaki K., and Chan F. K. (2013) Rip3: a molecular switch for necrosis and inflammation. Genes Dev. 27, 1640–1649 23913919 10.1101/gad.223321.113 PMC3744722

[54] Kaiser W. J., Upton J. W., Long A. B., Livingston-Rosanoff D., Daley-Bauer L. P., Hakem R., Caspary T., and Mocarski E. S. (2011) RIP3 mediates the embryonic lethality of caspase-8-deficient mice. Nature 471, 368–372 21368762 10.1038/nature09857 PMC3060292

[55] Arnold C. D., Gerlach D., Stelzer C., Boryń L̸. M., Rath M., and Stark A. (2013) Genome-wide quantitative enhancer activity maps identified by STARR-seq. Science 339, 1074–1077 23328393 10.1126/science.1232542

[56] Levine M., Cattoglio C., and Tjian R. (2014) Looping back to leap forward: transcription enters a new era. Cell 157, 13–25 24679523 10.1016/j.cell.2014.02.009 PMC4059561

[57] Georgiadi A., and Kersten S. (2012) Mechanisms of gene regulation by fatty acids. Adv. Nutr. 3, 127–134 22516720 10.3945/an.111.001602 PMC3648713

[58] Fatehi-Hassanabad Z., and Chan C. B. (2005) Transcriptional regulation of lipid metabolism by fatty acids: a key determinant of pancreatic β-cell function. Nutr. Metab. 2, 1 10.1186/1743-7075-2-1 PMC544854 15634355

[59] Grygiel-Górniak B. (2014) Peroxisome proliferator-activated receptors and their ligands: nutritional and clinical implications–a review. Nutr. J. 13, 17 24524207 10.1186/1475-2891-13-17 PMC3943808

[60] Kliewer S. A., Sundseth S. S., Jones S. A., Brown P. J., Wisely G. B., Koble C. S., Devchand P., Wahli W., Willson T. M., Lenhard J. M., and Lehmann J. M. (1997) Fatty acids and eicosanoids regulate gene expression through direct interactions with peroxisome proliferator-activated receptors α and γ. Proc. Natl. Acad. Sci. U.S.A. 94, 4318–4323 9113987 10.1073/pnas.94.9.4318 PMC20720

[61] Lo Verme J., Fu J., Astarita G., La Rana G., Russo R., Calignano A., and Piomelli D. (2005) The nuclear receptor peroxisome proliferator-activated receptor-α mediates the anti-inflammatory actions of palmitoylethanolamide. Mol. Pharmacol. 67, 15–19 15465922 10.1124/mol.104.006353

[62] Sethi S., Ziouzenkova O., Ni H., Wagner D. D., Plutzky J., and Mayadas T. N. (2002) Oxidized omega-3 fatty acids in fish oil inhibit leukocyte-endothelial interactions through activation of PPARα. Blood 100, 1340–1346 12149216 10.1182/blood-2002-01-0316

[63] Mishra A., Chaudhary A., and Sethi S. (2004) Oxidized omega-3 fatty acids inhibit NF-κB activation via a PPARα-dependent pathway. Arterioscler. Thromb. Vasc. Biol. 24, 1621–1627 15231516 10.1161/01.ATV.0000137191.02577.86

[64] Zhao Y., Calon F., Julien C., Winkler J. W., Petasis N. A., Lukiw W. J., and Bazan N. G. (2011) Docosahexaenoic acid-derived neuroprotectin D1 induces neuronal survival via secretase- and PPARγ-mediated mechanisms in Alzheimer's disease models. PLoS ONE 6, e15816 21246057 10.1371/journal.pone.0015816 PMC3016440

[65] Oeckinghaus A., Hayden M. S., and Ghosh S. (2011) Crosstalk in NF-κB signaling pathways. Nat. Immunol. 12, 695–708 21772278 10.1038/ni.2065

[66] Smale S. T. (2011) Hierarchies of NF-κB target-gene regulation. Nat. Immunol. 12, 689–694 21772277 10.1038/ni.2070 PMC3169328

[67] Ghosh S., and Hayden M. S. (2008) New regulators of NF-κB in inflammation. Nat. Rev. Immunol. 8, 837–848 18927578 10.1038/nri2423

[68] Jin H. S., Lee D. H., Kim D. H., Chung J. H., Lee S. J., and Lee T. H. (2009) cIAP1, cIAP2, and XIAP act cooperatively via nonredundant pathways to regulate genotoxic stress-induced nuclear factor-κB activation. Cancer Res. 69, 1782–1791 19223549 10.1158/0008-5472.CAN-08-2256

[69] Varfolomeev E., Goncharov T., Fedorova A. V., Dynek J. N., Zobel K., Deshayes K., Fairbrother W. J., and Vucic D. (2008) c-IAP1 and c-IAP2 are critical mediators of tumor necrosis factor α (TNFα)-induced NF-κB activation. J. Biol. Chem. 283, 24295–24299 18621737 10.1074/jbc.C800128200 PMC3259840

[70] Varfolomeev E., Blankenship J. W., Wayson S. M., Fedorova A. V., Kayagaki N., Garg P., Zobel K., Dynek J. N., Elliott L. O., Wallweber H. J., Flygare J. A., Fairbrother W. J., Deshayes K., Dixit V. M., and Vucic D. (2007) IAP antagonists induce autoubiquitination of c-IAPs, NF-κB activation, and TNFα-dependent apoptosis. Cell 131, 669–681 18022362 10.1016/j.cell.2007.10.030

[71] Conte D., Holcik M., Lefebvre C. A., Lacasse E., Picketts D. J., Wright K. E., and Korneluk R. G. (2006) Inhibitor of apoptosis protein cIAP2 is essential for lipopolysaccharide-induced macrophage survival. Mol. Cell. Biol. 26, 699–708 16382159 10.1128/MCB.26.2.699-708.2006 PMC1346893

[72] Bunting K., Rao S., Hardy K., Woltring D., Denyer G. S., Wang J., Gerondakis S., and Shannon M. F. (2007) Genome-wide analysis of gene expression in T cells to identify targets of the NF-κB transcription factor c-Rel. J. Immunol. 178, 7097–7109 17513759 10.4049/jimmunol.178.11.7097

[73] Grumont R. J., Richardson I. B., Gaff C., and Gerondakis S. (1993) rel/NF-κB nuclear complexes that bind κB sites in the murine c-rel promoter are required for constitutive c-rel transcription in B-cells. Cell Growth Differ. 4, 731–743 8241021

[74] Ballard D. W., Walker W. H., Doerre S., Sista P., Molitor J. A., Dixon E. P., Peffer N. J., Hannink M., and Greene W. C. (1990) The v-rel oncogene encodes a κB enhancer binding protein that inhibits NF-κB function. Cell 63, 803–814 2225078 10.1016/0092-8674(90)90146-6

[75] Hong S. Y., Yoon W. H., Park J. H., Kang S. G., Ahn J. H., and Lee T. H. (2000) Involvement of two NF-κB binding elements in tumor necrosis factor α-, CD40-, and Epstein-Barr virus latent membrane protein 1-mediated induction of the cellular inhibitor of apoptosis protein 2 gene. J. Biol. Chem. 275, 18022–18028 10751398 10.1074/jbc.M001202200

[76] Hannink M., and Temin H. (1990) Structure and autoregulation of the c-REL promoter. Oncogene 5, 1843–1850 2284104

[77] Viswanathan M., Yu M., Mendoza L., and Yunis J. (1996) Cloning and transcription factor-binding sites of the human c-REL proto-oncogene promoter. Gene 170, 271–276 8666258 10.1016/0378-1119(95)00773-3

[78] Nelson D. E., Ihekwaba A. E., Elliott M., Johnson J. R., Gibney C. A., Foreman B. E., Nelson G., See V., Horton C. A., Spiller D. G., Edwards S. W., McDowell H. P., Unitt J. F., Sullivan E., Grimley R., et al (2004) Oscillations in NF-κB signaling control the dynamics of gene expression. Science 306, 704–708 15499023 10.1126/science.1099962

[79] Ueberla K., Lu Y., Chung E., and Haseltine W. A. (1993) The NF-κB p65 promoter. J. Acquir. Immune Defic. Syndr. 6, 227–230 8450395

[80] Martin A. G., San-Antonio B., and Fresno M. (2001) Regulation of nuclear factor κB transactivation: implication of phosphatidylinositol 3-kinase and protein kinase Cζ in c-REL activation by tumor necrosis factor α. J. Biol. Chem. 276, 15840–15849 11278885 10.1074/jbc.M011313200

[81] Harris J., Olière S., Sharma S., Sun Q., Lin R., Hiscott J., and Grandvaux N. (2006) Nuclear accumulation of c-REL following C-terminal phosphorylation by TBK1/IKKϵ. J. Immunol. 177, 2527–2535 16888014 10.4049/jimmunol.177.4.2527

[82] Sánchez-Valdepeñas C., Martín A. G., Ramakrishnan P., Wallach D., and Fresno M. (2006) NF-κB-inducing kinase is involved in the activation of the CD28 responsive element through phosphorylation of c-REL and regulation of its transactivating activity. J. Immunol. 176, 4666–4674 16585559 10.4049/jimmunol.176.8.4666

[83] Antony R., Lukiw W. J., and Bazan N. G. (2010) Neuroprotectin D1 induces dephosphorylation of Bcl-xL in a PP2A-dependent manner during oxidative stress and promotes retinal pigment epithelial cell survival. J. Biol. Chem. 285, 18301–18308 20363734 10.1074/jbc.M109.095232 PMC2881755

[84] Faghiri Z., and Bazan N. (2010) PI3K/Akt and mTOR/p70S6K pathways mediate neuroprotectin D1-induced retinal pigment epithelial cell survival during oxidative stress-induced apoptosis. Exp. Eye Res. 90, 718–725 20230819 10.1016/j.exer.2010.03.002 PMC2873108

[85] Tamatani M., Che Y. H., Matsuzaki H., Ogawa S., Okado H., Miyake S., Mizuno T., and Tohyama M. (1999) Tumor necrosis factor induces Bcl-2 and Bcl-x expression through NFκB activation in primary hippocampal neurons. J. Biol. Chem. 274, 8531–8538 10085086 10.1074/jbc.274.13.8531

[86] Ahn H. J., Hernandez C. M., Levenson J. M., Lubin F. D., Liou H. C., and Sweatt J. D. (2008) c-Rel, an NF-κB family transcription factor, is required for hippocampal long-term synaptic plasticity and memory formation. Learn. Mem. 15, 539–549 18626097 10.1101/lm.866408 PMC2505322

[87] Sarnico I., Lanzillotta A., Boroni F., Benarese M., Alghisi M., Schwaninger M., Inta I., Battistin L., Spano P., and Pizzi M. (2009) NF-κB p50/RelA and c-REL-containing dimers: opposite regulators of neuron vulnerability to ischaemia. J. Neurochem. 108, 475–485 19094066 10.1111/j.1471-4159.2008.05783.x

[88] Mokhtari D., Barbu A., Mehmeti I., Vercamer C., and Welsh N. (2009) Overexpression of the nuclear factor-κB subunit c-REL protects against human islet cell death *in vitro*. Am. J. Physiol. Endocrinol. Metab. 297, E1067–E1077 19706790 10.1152/ajpendo.00212.2009

[89] Gilmore T. D., and Gerondakis S. (2011) The c-REL transcription factor in development and disease. Genes Cancer 2, 695–711 22207895 10.1177/1947601911421925 PMC3218406

[90] Sarnico I., Lanzillotta A., Benarese M., Alghisi M., Baiguera C., Battistin L., Spano P., and Pizzi M. (2009) NF-κB dimers in the regulation of neuronal survival. Int. Rev. Neurobiol. 85, 351–362 19607980 10.1016/S0074-7742(09)85024-1

[91] Zhang J., Wang C., Shen Y., Chen N., Wang L., Liang L., Guo T., Yin X., Ma Z., Zhang B., and Yang L. (2016) A mutation in ADIPOR1 causes nonsyndromic autosomal dominant retinitis pigmentosa. Hum. Genet. 135, 1375–1387 27655171 10.1007/s00439-016-1730-2

